# Clinical and genetic characteristics of congenital sideroblastic anemia: comparison with myelodysplastic syndrome with ring sideroblast (MDS-RS)

**DOI:** 10.1007/s00277-012-1564-5

**Published:** 2012-09-16

**Authors:** Rie Ohba, Kazumichi Furuyama, Kenichi Yoshida, Tohru Fujiwara, Noriko Fukuhara, Yasushi Onishi, Atsushi Manabe, Etsuro Ito, Keiya Ozawa, Seiji Kojima, Seishi Ogawa, Hideo Harigae

**Affiliations:** 1 Department of Hematology and Rheumatology, Tohoku University Graduate School of Medicine, 1-1 Seiryo-machi, Aoba-ku, Sendai, 980-8574 Japan; 2 Department of Molecular Biology and Applied Physiology, Tohoku University Graduate School of Medicine, Sendai, Japan; 3 Cancer Genomics Project, Graduate School of Medicine, The University of Tokyo, Tokyo, Japan; 4 Department of Molecular Hematology/Oncology, Tohoku University Graduate School of Medicine, Sendai, Japan; 5 Department of Pediatrics, St Luke’s International Hospital, Hirosaki University Graduate School of Medicine, Hirosaki, Japan; 6 Department of Pediatrics, Hirosaki University Graduate School of Medicine, Hirosaki, Japan; 7 Division of Hematology, Jichi Medical University, Shimotsuke, Japan; 8 Department of Pediatrics, Nagoya University Graduate School of Medicine, Nagoya, Japan

**Keywords:** Congenital sideroblastic anemia, Myelodysplastic syndrome, ALAS2

## Abstract

Sideroblastic anemia is characterized by anemia with the emergence of ring sideroblasts in the bone marrow. There are two forms of sideroblastic anemia, i.e., congenital sideroblastic anemia (CSA) and acquired sideroblastic anemia. In order to clarify the pathophysiology of sideroblastic anemia, a nationwide survey consisting of clinical and molecular genetic analysis was performed in Japan. As of January 31, 2012, data of 137 cases of sideroblastic anemia, including 72 cases of myelodysplastic syndrome (MDS)–refractory cytopenia with multilineage dysplasia (RCMD), 47 cases of MDS–refractory anemia with ring sideroblasts (RARS), and 18 cases of CSA, have been collected. Hemoglobin and MCV level in CSA are significantly lower than those of MDS, whereas serum iron level in CSA is significantly higher than those of MDS. Of 14 CSA for which DNA was available for genetic analysis, 10 cases were diagnosed as X-linked sideroblastic anemia due to *ALAS2* gene mutation. The mutation of *SF3B1* gene, which was frequently mutated in MDS-RS, was not detected in CSA patients. Together with the difference of clinical data, it is suggested that genetic background, which is responsible for the development of CSA, is different from that of MDS-RS.

**Electronic supplementary material:**

The online version of this article (doi:10.1007/s00277-012-1564-5) contains supplementary material, which is available to authorized users.

## Introduction

Sideroblastic anemia is characterized by anemia with the emergence of ring sideroblasts in the bone marrow. Ring sideroblasts are formed by the irregular accumulation of iron in mitochondria. There are two forms of sideroblastic anemia i.e., congenital sideroblastic anemia (CSA) and acquired sideroblastic anemia. Most of acquired sideroblastic anemia cases were included in myelodysplastic syndrome (MDS). To date, mutations of genes involved in heme biosynthesis, Fe–S cluster biogenesis, or the biology of mitochondria have been reported in CSA [1–5]. Impaired function of these genes is speculated to result in disutilization of iron, leading to accumulation of iron in mitochondria. Acquired sideroblastic anemia in MDS is categorized either as refractory cytopenia with multilineage dysplasia (RCMD) or refractory anemia with ring sideroblasts (RARS) depending on the level of dysplasia. In contrast CSA, mechanism of forming ring sideroblasts in MDS is not clarified, although it was recently suggested that the mutations of splicing pathway are involved in the pathogenesis of MDS [6]. It is possible that there is a common mechanism between CSA and MDS; however, mutations in genes, which are responsible for development of the CSA, have not been identified in MDS.

The most common form of CSA is X-linked sideroblastic anemia (XLSA), which is caused by mutation of erythroid-specific 5-aminolevulinate synthase (*ALAS2*), the first enzyme of heme synthesis in erythroid cells [7–10]. More than half of the patients with XLSA respond to the administration of pyridoxine [vitamin B6 (Vit.B6)], or pyridoxal 5-phosphate (PLP), which is the coenzyme of *ALAS2* [11]. In XLSA, adult onset cases have been reported [12, 13]; therefore, it is possible that some cases of CSA may be misdiagnosed as MDS, especially RARS. However, the clinical and pathological features of congenital and acquired sideroblastic anemia have not been fully clarified because there have been no comprehensive studies, including clinical and genetic analyses, focusing on sideroblastic anemia.

Here, we performed a nationwide survey of sideroblastic anemia in Japan to investigate the epidemiology and pathogenesis of this disease. The difference of clinical data and results of genetic analysis suggest that genetic background, which is responsible for the development of CSA, is distinct from that of MDS-RS.

## Materials and methods

### Data acquisition

This study consisted of three investigations. First, patients with sideroblastic anemia were searched by questionnaire sent to hospitals with hematology department (493 hospitals) and pediatric hematology department (593 hospitals) asking for information about patients diagnosed as sideroblastic anemia (first investigation) over the past 10 years. Next, detailed clinical data of sideroblastic anemia patients were collected from the hospital based on responses to the first investigation (second investigation). Survey items were age of onset, gender, family history, hematological and biochemical findings, treatment, and cause of death. Then, genetic analysis of patients, who were diagnosed as CSA and MDS without chromosomal anomaly, was performed in cases for which genome sample was available (third investigation).

This study was approved by the ethics committee of Tohoku University Graduate School of Medicine, the center responsible for clinical and genetic analysis. Informed consent for the genetic analysis was obtained in all cases.

### Diagnostic procedure

Ring sideroblasts were defined following the 2001 World Health Organization (WHO) classification. Sideroblastic anemia patients were diagnosed in the respective institutions. In all cases, bone marrow smears were investigated, and at least 15 % ring sideroblasts were confirmed by iron staining. Furthermore, diagnosis for RARS was made when dysplasia restricted to erythroid lineage in bone marrow was recognized. Diagnosis for RCMD was made when there is multilineage dysplasia. Thereafter, in the present study, RCMD correspond to refractory cytopenia with multilineage dysplasia and ringed sideroblasts (RCMD-RS) of the 2001 WHO classification. Diagnosis for CSA was made when the patient had a family history or the disease onset during infancy, or fulfilled the characteristic features of XLSA, such as onset at a young age, microcytic anemia, and responsiveness to Vit.B6.

### Genetic analysis of patients with sideroblastic anemia

In the genetic analysis, mutations in *ALAS2*, *SLC25A38*, *GLRX5*, *ABCB7*, *PUS1*, and *SLC19A2,* which are known to be responsible for CSA, were examined in 14 cases of CSA and 10 cases of MDS. In addition, *SF3B1*, which was very recently reported to be mutated in sideroblastic anemia in MDS at a high incidence, were analyzed as well. Mutation analysis for the *ALAS2* gene was performed first in all candidates, and then the analysis proceeded to the other genes if no mutations in *ALAS2* were detected. For mutation analysis of *ALAS2*, genomic DNA was extracted from the proband’s peripheral blood using QIAamp DNA blood midi kit (QIAGEN, Valencia, CA, USA). The proximal promoter region [14], erythroid enhancer in intron 8 [15], and all exons and exon–intron boundaries of the *ALAS2* gene were amplified using ExTaq DNA polymerase (Takara Bio, Shiga, Japan) [16]. Amplified products were purified using a QIAquick gel extraction kit (QIAGEN) after agarose gel electrophoresis. They were then subjected to direct sequencing analysis using BigDye Terminator Cycle sequencing kit v1.1 with an ABI3100 genetic analyzer (Life Technologies Corp., Carlsbad, CA, USA). Mutation of the gene was confirmed by repeated polymerase chain reaction (PCR) followed by direct sequencing analysis. Genes other than *ALAS2* were sequenced by Hiseq2000® [6]. Briefly, genomic DNA was amplified using REPLI-g mini kit® (QIAGEN Science). After adjusting the concentration of amplified DNA, DNA from consecutive 12 samples was combined into one DNA pool, and the entire coding sequences were amplified by primers to which *Not*I linker was attached. The products were digested with *Not*I, and ligated with T4 ligase. Then, DNA was sonicated into ~200-bp fragments, and sequencing libraries were generated. Libraries were subjected to deep sequencing on Hiseq2000®. Sequencing data was analyzes as described previously. Detected mutations were validated by direct sequence.

### Analysis of enzymatic activity of recombinant ALAS2 protein

For preparing recombinant ALAS2 proteins, complementary DNA (cDNA) encoding mature human ALAS2 protein was amplified using a following primer set (5′-GGTGGTCATATGATCCACCTTAAGGCAACAAAGG-3′and 5′-GGCATAGGTGGTGACATACTG-3′). The amplified cDNA was then treated with *Nde*I restriction enzyme and was cloned between *Nde*I and blunt-ended *Sap*I site of pTXB1 plasmid (New England Biolabs, Ipswich, MA, USA), resulting in pTXB-AEm. From this plasmid, mature ALAS2 protein was expressed as an inducible fusion protein with Intein and chitin-binding domain in *E. coli*. To obtain the mutant protein, the identified mutation was introduced into pTXB-AEm using PrimeStar Max site-directed mutagenesis kit (Takara Bio, Shiga, Japan). For expression and purification of wild-type and mutant ALAS2 proteins, *E. coli* BL21 (DE3) was transformed with each plasmid. The induction and purification of the recombinant proteins were performed using Impact system (New England Biolabs) according to manufacturer’s instruction. Briefly, each recombinant protein was induced in *E. coli* with 0.1 mM IPTG at 25 °C for overnight. Then, cells were resuspended with lysis buffer (20 mM Tris–HCl pH 8.5, 500 mM NaCl, 1 mM EDTA, 0.1 % Triton X-100, 1 mM PMSF, 1 μg/ml of antipain, pepstatin, and leupeptin). After the sonication and centrifugation, cleared cell lysates were incubated with chitin beads for 1 h at 4 °C, then washed with wash buffer (20 mM Tris–HCl pH 8.5, 500 mM NaCl, 1 mM EDTA, and 0.1 % Triton X-100). Tag-free recombinant mature ALAS2 protein was obtained by on-column cleavage with 50 mM DTT in wash buffer at room temperature for 16 h. After the elution from the column, protein concentration was determined using Bio-Rad Protein assay reagent (Bio-Rad Laboratories, Inc., Hercules, CA, USA). The ALAS activity of each recombinant protein was measured in vitro, as described previously [8].

### Statistical analysis

Results are presented as means ± SD with the exception of the age of onset, which is expressed as the median. Statistical analysis was performed using Student’s *t* test, and *P* < 0.05 was taken to indicate statistical significance.

## Results

### Epidemiology of sideroblastic anemia

As of 31 January 2012, detailed data for 148 sideroblastic anemia, including MDS and secondary sideroblastic anemia, patients have been collected. Excluding 10 cases of refractory anemia with excess blasts (RAEB) and one case of sideroblastic anemia due to alcohol, the remaining 137 cases were classified as 18 cases of CSA, 47 cases of RARS, and 72 cases of RCMD. Of 18 CSA cases, 7 were already confirmed to be XLSA due to mutation of *ALAS2* before registration in this study, and the others were diagnosed as CSA based on family history or clinical findings, including responsiveness to Vit.B6 treatment. Clinical findings and family history, which suggest the porphyria, were not observed in any CSA patients.

### Analysis of the pathology of congenital sideroblastic anemia

Laboratory data of CSA, RARS, and RCMD are shown in Tables 1 and 2. Median age at onset of CSA was younger than those of RARS and RCMD (19, 72.5, and 71 years old, respectively). Hemoglobin and mean corpuscular volume (MCV) values of CSA were significantly lower than those of RARS and RCMD cases (7.1 g/dl and 69.0 fl, 8.7 g/dl and 106.8 fl; and 8.3 g/dl and 106.5 fl, respectively). Serum iron level in CSA was significantly higher than that in RARS or RCMD (210.7, 162.8, and 171.1 μg/dl, respectively). These data have possibilities of reflecting the states of the iron over-loaded of CSA; however, as serum iron concentration is very instable and depends from different factors, this finding should be carefully evaluated.

**Table 1:** Clinical data of CSA, RARS, and RCMD (1)

	CSA (*n* = 18)	RARS (*n* = 47)	RCMD (*n* = 72)	*p*-value (between CSA and RARS)	*p*-value (between CSA and RCMD)
Gender					
Male	17	33	44		
Female	1	14	28		
Median age at onset (year)	19.0 (±20.2)	72.5 (±10.4)	71.0 (±13.0)	<0.01	<0.01
White blood cells (/μl)	5547 (±2022)	4741 (±2561)	4105 (±1847)	0.24	<0.01
Red blood cells (×10^4^/μl)	383.4 (±100.0)	245.6 (±45.6)	239.4 (±56.4)	<0.01	<0.01
Hemoglobin (g/dl)	7.1 (±2.21)	8.7 (±1.7)	8.3 (±1.8)	<0.01	0.02
Mean corpuscular volume (fl)	69.0 (±11.6)	106.8 (±9.0)	106.5 (±9.2)	<0.01	<0.01
Platelet (×10^4^/µl)	28.5 (±12.62)	25.9 (±15.5)	23.9 (±24.1)	0.53	0.44
Reticulocyte (‰)	12.1 (±10.9)	17.7 (±10.8)	21.5 (±20.1)	0.07	0.05

**Table 2:** Clinical data of CSA, RARS, and RCMD (2)

	CSA (*n* = 18)	RARS (*n* = 47)	RCMD (*n* = 72)	*p*-value (between CSA and RARS)	*p*-value (between CSA and RCMD)
Total bilirubin (mg/dl)	1.1 (±0.8)	1.3 (±0.9)	1.1 (±0.7)	0.47	0.78
AST (GOT) (IU/l)	33.0 (±24.3)	24.9 (±11.7)	27.9 (±20.8)	0.08	0.38
LDH (IU/l)	218.3 (±98.9)	263.5 (±119.2)	246.1 (±97.7)	0.16	0.28
CRP (mg/dl)	0.13 (±0.15)	0.40 (±1.16)	1.17 (±3.81)	0.37	0.30
Serum iron (mg/dl)	210.7 (±77.6)	162.8 (±73.6)	171.1 (±66.2)	0.03	0.04
UIBC (mg/dl)	80.4 (±113.6)	102.4 (±82.7)	79.9 (±60.7)	0.48	0.93
Ferritin (ng/ml)	1239.8 (±1306.8)	743.4 (±815.3)	804.3 (±990.2)	0.08	0.13

When iron-related laboratory data were examined in transfusion independent cases (CSA, 13; RARS, 26; RCMD, 34), Serum iron level in CSA was tended to be higher than that in RARS or RCMD (210.6, 180.3, and 166.6 μg/dl, respectively), although the difference was not significant (*p* = 0.07, data not shown). Serum ferritin level in CSA, RARS and RCMD were elevated in these transfusion independent cases (1,087.9, 898.1, and 732.2 ng/ml, respectively), suggesting that most of sideroblastic anemia patients were iron-overloaded before transfusion. There were no significant differences in other biochemical data among the three groups.

### Chromosomal abnormalities of MDS

**Fig. 1:**
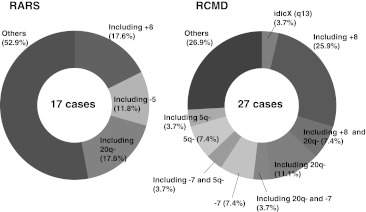
Chromosomal abnormalities in RARS and RCMD. Data of chromosomal analysis in RARS and RCMD are shown. +8 was most common both in RARS and RCMD. -7 was only seen in RCMD

Data regarding cytogenetic abnormalities were available for all RARS patients and for 68 of 72 RCMD patients. Figure 1 shows the cytogenetic findings of RARS and RCMD. In RARS cases, chromosomal abnormalities were found in 17 patients (36.2 %). Abnormalities consisted of abnormality including +8 (3 cases), complex abnormality with deletion 5 (2 cases), and complex abnormality with 20q− (3 cases). Chromosomal abnormalities in RCMD were found in bone marrow samples from 27 RCMD patients (39.7 %). Abnormality including +8 was detected in nine cases (33.3 %) and abnormality of idic (X) (q13), associated with the *ABCB7* gene [17], was found in one case. In addition, −7, which was not identified in RARS, was identified in four RCMD patients (14.8 %).

### Treatment and outcome

Analysis of the available data regarding treatment indicated that 17 of 47 RARS cases and 26 of 72 RCMD cases were administered Vit.B6 (data not shown). The effectiveness was judged according to the criteria of IWG [18], and one RARS patient obtained a major response, and three RARS patients and one RCMD patient obtained minor responses. Thus, 4 of 17 RARS patients and 1 of 26 RCMD patients responded to Vit.B6 treatment. However, improvement of Hb was not sustained in two RARS patients; Hb level gradually returned to or dropped below the pretreatment level. Therefore, Vit.B6 treatment may not be effective for MDS, or the effect if any may be very limited. The clinical outcomes of patients are shown in Supplemental Table 1. The median follow-up from the time of diagnosis in CSA patients was 30.5 months, and two patients died due to sepsis (one case) and cardiac failure (one case). One patient who died due to cardiac failure was heavily iron overloaded as defined by serum ferritin level, suggesting that cardiac complications may be caused by hemochromatosis. The median follow-up from the time of diagnosis in RARS patients was 23 months, and 6 patients (12.8 %) died due to pneumonia (two cases), evolution to leukemia (one case), and others (three cases). The median follow-up from the time of diagnosis in RCMD patients was 19.5 months, and 20 patients (27.8 %) died due to pneumonia (7 cases), cardiac failure (3 cases), evolution to leukemia (2 cases), sepsis (1 case), and others (7 cases). These results suggest that the prognosis of RCMD is worse than that of RARS.

### Gene analysis of congenital sideroblastic anemia

Eighteen CSA patients were candidates for gene analysis; however, mutation analysis for genes responsible for CSA was not performed in four patients. One patient was diagnosed as having PMPS based on clinical findings, and DNA samples were not available for the remaining three patients. Therefore, gene analysis was performed in 14 of 18 CSA patients. Ten of these 14 patients were diagnosed as XLSA due to *ALAS2* mutation. Table 3 summarizes the results of gene analysis in XLSA. Case 2 (R411C), case 4 (D190V), case 6 (M567I), and case 7 (V562A) were reported previously [19–21]. Since amino acid substitution at Arg170, 411, and 452 were observed in plural patients, there are hot spots of mutation of *ALAS2* gene.

**Table 3:** Congenital sideroblastic anemia (XLSA)

Case number	Age at diagnosis (y.o.)	Gender	Position of *ALAS2* mutation	*SF3B1* mutation	Hb at onset (g/dl)	MCV at onset (fl)	Increment of Hb by Vit.B6 treatment (g/dl)	In vitro enzymatic activity of mutant protein^a^	
								Without PLP	With PLP
1	0	M	R170C	N/D	4.8	52.5	1.7	64.1 %	72.5 %^b^
2	20	M	R411C	N/D	4.8	52.5	5.2	11.9 %	25.1 % [19]
3	68	M	R452C	–	6.0	67.3	No effect	99.9 %	94.0 % [21]
4	17	M	D190V	N/D	8.9	66.9	No effect	98.6 %	98.5 % [20]
5	36	M	R452C	–	7.4	70.0	No effect	99.9 %	94.0 % [21]
6	36	M	M567I	N/D	6.5	64.4	3.4	38.1 %	25.2 % [21]
7	14	M	V562A	–	8.1	61.2	4.7	150.6 %	116.9 % [21]
8	31	M	R170L	–	4.1	50.8	8.1	31.1 %	60.8 %^b^
9	3	M	R411C	–	5.4	54.4	2.9	11.9 %	25.1 % [19]
10	62	M	R170L	N/D	8.0	73.9	No effect	31.1 %	60.8 %^b^

^a^% of WT

^b^Present study

**Fig. 2:**
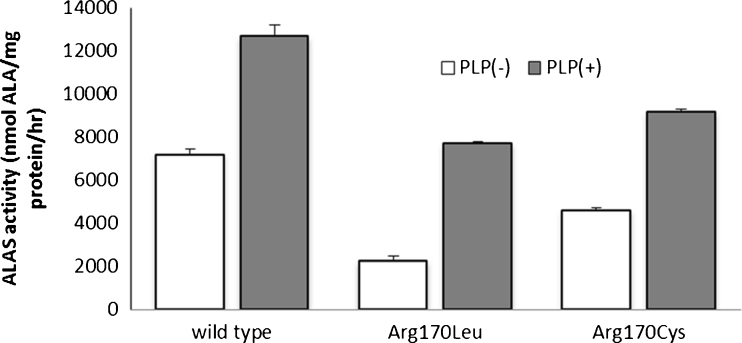
Enzymatic activity of mutant ALAS2 proteins. Enzymatic activity of wild-type and mutant ALAS2 proteins was measured as described in Materials and Methods. Both of R170L and R170C ALAS2 mutant proteins showed decreased enzymatic activity; however, the activity was partially restored by the addition of PLP

Patient with D190V (case 4), R170L (Case 10) and two patients with R452C (cases 3 and 5) did not respond to Vit.B6 treatment, whereas six patients responded to Vit.B6 treatment, although the increment of hemoglobin varied from 1.7 to 8.1 g. Interestingly, case 8 responded to Vit.B6 treatment, whereas case 10 did not, although both of them harbor the same mutation, R170L. Therefore, the activity of R170L mutant proteins was examined to determine the property, especially the Vit.B6 responsiveness. The enzymatic activities of wild type and R170L mutant protein were 7,193 ± 138 nmol ALA/mg protein/h and 2,240 ± 145 nmol ALA/mg protein/h, respectively, in the absence of PLP (Fig. 2). With an excess amount of PLP (100 μM) in the assay mixture, higher enzymatic activities were obtained with wild-type and mutant proteins (12,662 ± 311 nmol ALA/mg protein/h and 7,700 ± 49 nmol ALA/mg protein/h, respectively) (Fig. 2). In addition, the enzymatic activity of R170C, which is another substitution at Arg170 found in this study, was also examined. As shown in Fig. 2, The enzymatic activity of mutant protein was significantly lower than wild-type protein without PLP (4,612 ± 87 nmol ALA/mg protein/h vs 7,193 ± 138 nmol ALA/mg protein/h), and the activity was restored by addition of excess amount of PLP (100 μM) in the assay mixture. These in vitro data suggest that amino acid substitution at Arg 170, at least R170L and R170C, results in the decrease in enzymatic activity, but the decrease can be recovered by excess amount of PLP. The enzymatic activity of mutant proteins, which were identified in this study, is summarized in Table 3. The enzymatic activities of R411C, D190V, M567I, and V562A were referred from previous reports [19–21]. The levels of activity and PLP responsiveness in vitro were not correlated with clinical responsiveness to PLP in some cases. It is possible that the variety of mechanisms, such as the decrease in enzymatic activity of mutant ALAS2 protein, the changes of amount of *ALAS2* transcript, and physiological and environmental status of the patients, are responsible for the development of the disease.

Data for CSA patients other than XLSA are summarized in Table 4. Case 15 was diagnosed as PMPS. Gene analysis was not performed for cases 16 and 17; however, XLSA was strongly suspected because these patients were male and had microcytic anemia that was responsive to Vit.B6 treatment. *ALAS2* mutations were not identified in cases 11, 12, 13, and 14. Therefore, mutations of *SLC25A38*, *GLRX5*, *ABCB7*, *PUS1*, *SLC19A2*, and *SF3B1* were examined in these cases; however, no mutations were identified in these cases. In contrast to other cases, case 18 was female and showed normocytic anemia. She was diagnosed with CSA due to family history; however, gene mutation analysis was not performed because DNA samples were not available. *SF3B1* gene mutation was examined in nine cases including five XLSA, however, no mutation was identified (Tables 3 and 4). On the other hand, *SF3B1* gene mutation was frequently detected in MDS-RS (Table 5).

**Table 4:** Congenital sideroblastic anemia (other than XLSA)

Case number	Age at diag (y.o.)	Gender	Family history	Gene mutation							Hb (g/dl)	MCV (fl)	Response to Vit.B6
				*ALAS2*	*SLC25A38*	*GLRX5*	*ABCB7*	*SLC19A2*	*PUS1*	*SF3B1*			
11	19	M	−	−	−	−	−	−	−	−	7.8	73.9	−
12	4	M	−	−	−	−	−	−	−	−	6.6	73.6	−
13	0	M	+	−	−	−	−	−	−	−	3.9	65.0	−
14	20	M	+	−	−	−	−	−	−	−	7.6	82.0	+
15	0	M	−	N/D	N/D	N/D	N/D	N/D	N/D	N/D	6.8	88.1	N/D^a^
16	32	M	−	N/D	N/D	N/D	N/D	N/D	N/D	N/D	11.2	69	+
17	36	M	−	N/D	N/D	N/D	N/D	N/D	N/D	N/D	10.8	67.3	+
18	18	F	+	N/D	N/D	N/D	N/D	N/D	N/D	N/D	9.3	96.2	+

*N/D* not done

^a^Vit.B6 was not administered due to PMPS

**Table 5:** Mutation of *SF3B1 *gene in MDS-RS

Case number	Diagnosis	Age at diagnosis (y.o.)	Gender	Chromosome anomaly	position of *SF3B1* mutation
1	RARS	82	M	–	E622D
2	RARS	57	M	–	N626S
3	RARS	60	M	Complex karyotype, including +8	K700E
4	RARS	60	M	–	K700E
5	RARS	73	F	–	No mutation
6	RARS	74	F	–	H662Q
7	RARS	76	M	–	K700E
8	RARS	67	F	–	K700E
9	RARS	66	M	–	K666E
10	RCMD	50	F	–	No mutation

(–) normal karyotype

## Discussion

Because of its rarity, there have been few clinical and pathological investigations focusing on sideroblastic anemia. This study was performed to investigate the epidemiological and pathological characteristics of sideroblastic anemia. Based on the data of 137 patients, it was revealed that hemoglobin level in CSA was significantly lower than those seen in MDS, and serum iron level was higher in CSA compared to MDS. These results revealed that anemia in CSA is more severe than that in MDS at onset, although significant cases improved by Vit.B6 treatment. Reflecting the high incidence of XLSA in CSA, MCV level was significantly lower in CSA than MDS. These findings suggest that CSA should be strongly suspected rather than MDS, at least in Japan, in male patients exhibiting microcytic anemia and an elevated serum iron level.

MDS-RCMD is the most common form of acquired sideroblastic anemia. Chromosomal abnormalities were observed in 39.7 % of RCMD cases and 36.2 % of RARS cases. The types of chromosomal abnormality frequently observed in RCMD and RARS did not differ from those reported previously, such as +8, −7, 20q− and −5. Among them, +8 was observed in nine cases of RCMD (33.3 %). As the frequency of +8 in MDS was reported to be 6.5–16.7 %, +8 appeared to be more common in RCMD. In addition, −7 was identified in four patients with RCMD (14.8 %), whereas it was not identified in RARS. This difference may be related to the poor prognosis of RCMD.

Regarding the responsiveness to pyridoxine treatment among XLSA, 6 of 10 cases responded to Vit.B6 treatment in this study, although the magnitude of response varied among individuals. Thus, as the benefit of treatment of Vit.B6 for XLSA is obvious, a precise diagnosis of XLSA is important. As late-onset XLSA cases have been reported and two patients over 60 years old were found in this study, genetic analysis in sideroblastic anemia patients with microcytic anemia is essential regardless of age.

Focusing on *ALAS2* mutation in XLSA, two patients with the same mutation (c.509G>T), which results in R170L, showed distinct responses to Vit.B6. Edgar et al. [22] reported a Vit.B6 responsive pedigree with XLSA carrying the p.R170L mutation of ALAS2 gene. Furthermore, the crystal structure analysis of ALAS from *Rhodobacter capsulatus* [23] suggests that a missense mutation at Arg170 destabilizes PLP binding, which might be partially restored with excess amounts of PLP. Together with the findings of biochemical analysis in this study, it is strongly suggested that R170L mutation causes pyridoxine-responsive XLSA. However, in consistent with the data of in vitro analysis and clinical course of other R170L patients, case 10 was unresponsive to Vit.B6 treatment. Thus, onset and severity of the disease may be defined by not only the type of mutation but also the environmental and physiological status of the patients. This speculation may be supported by the results that there is a discrepancy between in vitro and in vivo response to Vit.B6 in some cases (Table 3).

The high incidence of XLSA among CSA in the present study was consistent with a previous report in the USA. Bergmann et al. [24] reported genetic analysis of CSA in the USA. In this study, mutations of *ALAS2*, *SLC25A38*, mitochondria DNA, and *PUS1*, were identified in 37, 15, 2.5, and 2.5 % of CSA cases, respectively. The most significant difference from our study was that mutations of the *SLC25A38* gene were frequently found in the USA. Since *SLC25A38* is thought to be a transporter of glycine, which is a substrate for ALAS2 in the first step of heme synthesis, the pathology of CSA due to mutation of this gene is similar to that of XLSA. Therefore, CSA patients with microcytic anemia, in whom mutations of *ALAS2* gene were not identified, were expected to harbor *SLC25A38* mutation; however, it was not detectable in this study. To date, it has not been reported in Asia, although mutation of the *SLC25A38* gene has been widely reported in the USA, Canada, and Europe. Together with the results of the present study, it is suggested that the causative genes of CSA differ among races and regions.

Recently, mutations of genes involved in splicing machinery were reported in MDS [6]. Among them, *SF3B1*, which is a component of the U2-small nuclear ribonucleoprotein (U2-snRNP) complex [25], was found to be highly mutated in MDS with ring sideroblasts [6]. In this study, *SF3B1* mutation was examined in nine cases of CSA; however, its mutation was not detectable in CSA. These findings suggest that the mechanism for sideroblasts formation may be different between CSA and MDS.

In conclusion, our data showed that XLSA is the most frequent type of CSA; however, onset and severity of the disease may be affected by the environmental and physiological status of the patients. The data, including clinical and genetic analysis, further suggest that genetic background is different between CSA and MDS.

## Electronic supplementary material

Below is the link to the electronic supplementary material.

ESM 1 (PDF 10 kb)
