# Supplementary material for: Clinical and genetic characteristics of congenital sideroblastic anemia: comparison with myelodysplastic syndrome with ring sideroblast (MDS-RS)
Source: Ann Hematol. 2012 Sep 16;92(1):1–9. doi: 10.1007/s00277-012-1564-5 (PMC3536986; doi:10.1007/s00277-012-1564-5)
Supplement: Supplementary file 1 — (PDF 10 kb) [file 277_2012_1564_MOESM1_ESM.pdf]

Supplemental Table 1.
Clinical outcome of CSA, RARS, and RCMD

	CSA (n=18)	RARS (n=47)	RCMD (n=72)
Alive (cases)	16	41	52
Death (cases)	2	6	20
Median months from the time of diagnosis	30.5	23.0	19.5
Cause of death (cases)	Cardiac failure 1<br>Sepsis 1	Pneumonia 2<br>Evolution to leukemia 1<br>Others 3	Pneumonia 7<br>Cardiac failure 3<br>Evolution to leukemia 2<br>Sepsis 1<br>Chronic GVHD after allo-SCT* 2<br>Others 5

* allo-SCT: allogeneic stem cell transplantation
